# Preparation and characterization of emamectin benzoate nanoformulations based on colloidal delivery systems and use in controlling *Plutella xylostella* (L.) (*Lepidoptera*: *Plutellidae*)

**DOI:** 10.1039/c8ra01913d

**Published:** 2018-04-26

**Authors:** Ali Shoaib, Muhammad Waqas, Asem Elabasy, Xinlai Cheng, Qianqian Zhang, Zuhua Shi

**Affiliations:** Key Laboratory of Molecular Biology of Crop Pathogens and Insects, Ministry of Agriculture, Institute of Insect Sciences, Zhejiang University 866 Yuhangtang Road Hangzhou 310058 China zhshi@zju.edu.cn; Department of Pesticides, Plant Protection Research Institute, Agricultural Research Centre Cairo Egypt

## Abstract

Colloidal delivery systems have been widely used as carriers for controlled delivery of pesticides to improve the efficacy and photostability of natural and semi-synthetic pesticides. In this study, we have synthesized emamectin benzoate nanoformulations (EB + NFs) depending on polymeric nanocapsules (PNC) and two types of the nanosilica, mesoporous nanosilica (MCM-48) and silicon dioxide nanoparticles (SNPs) as carriers for the emamectin benzoate (EB). The fabricated nanoformulations were characterized by using X-ray diffraction analysis, Fourier transform infrared spectroscopy, particle size, zeta potential, morphology, absolute recovery (AR), entrapment efficiency (EE), UV stability and release kinetics. The obtained results showed that the carriers had a remarkable loading ability for EB and improved the EB photostability. The EE% of nanoformulations were 92.84%, 87.45% and 71.19% for emamectin benzoate polymeric nanocapsules (EB + PNC), emamectin benzoate SNPs (EB + SNPs) and emamectin benzoate MCM-48 (EB + MCM-48) respectively. The insecticidal activity of EB + NFs against *Plutella xylostella* showed that the EB + SNPs was more effective than other EB + NFs and EB alone. The LC_50_ values were 0.18, 4.03, 8.49 and 11.06 mg L^−1^ for EB + SNPs, EB + MCM-48, EB + PNC and EB respectively. The obtained results suggest the colloidal delivery systems that used in this study could improve the efficacy and photostability for EB, and they are able to overcome the disadvantage of the natural and semi-synthetic pesticides such as environmental sensitivity and to increase the efficacy of pesticides, which eventually leads to reduce the dosage of pesticides needed, reducing the number of applications required in comparison to conventional formulations.

## Abstract 2

Colloidal delivery systems have been widely used as carriers for controlled delivery of pesticides to improve the efficacy and photostability of natural and semi-synthetic pesticides.

## Graphical abstract

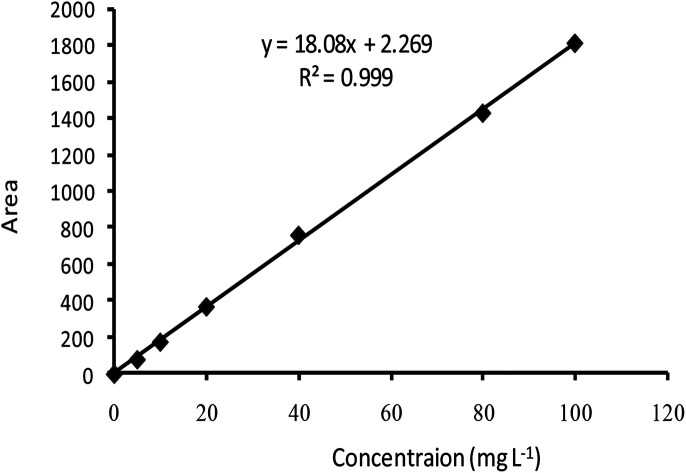

## 1. Introduction

Herbivorous insects are very destructive pests for the important crops and their production. These insects cause damage by feeding on seedlings, germinating seeds and flowers. The chemical pesticides are useful for protecting the crops from insect damage during the growing season. However, indiscriminate use of them has led to several environmental problems, including serious health hazards to humans and animals, development of insecticide resistance, destruction of beneficial insects and accumulation of pesticide residues in different environmental compartments.

In the recent years, natural and semi-synthetic pesticides have gained interest as a promising alternative to conventional pesticides for pest insect control,^1^ but these pesticides exist obviously some disadvantages, including low activity and short persistent under various environmental conditions such as sunlight, humidity and rainfall. Emamectin benzoate (EB) is a semi-synthetic derivative of abamectin of the avermectin family of 16-membered macrocylic lactones. This epi-methyl amino derivative showed increased effectiveness against a broad spectrum of lepidopterous and coleopterous pests with application rates in active ingredient (a.i.) ranging between 8.4–16.8 g ha^−1^.^2,3^ Unfortunately, the avermectin compounds are degraded rapidly from the environment after application. The binding compounds to the soil are rapidly decomposed by the soil microorganisms after fast photolysis on plant surfaces. Additionally, the current commercial formulations of EB are sensitive to the light and temperature. These problems limit the use of EB in agriculture sector because an insecticide should persist in the field for enough time to ensure adequate control of pests.^2^ Therefore, it is an urgent need to produce formulations that could meet the requirements of high efficiency and prolonged protection.

Colloidal delivery systems such as polymeric nanocapsules (PNC) and polymeric nanospheres (PNS) have been used to overcome these disadvantages and to improve the insecticidal properties according to the principle of controlled release formulations (CRFs). CRFs have the ability to reduce the environmental hazards of excessive use of pesticides around the world to protect crops. In many cases the use of CRFs could reduce the total applied amount of pesticide a.i. by reducing the concentration and time of application leading to reduce economic cost as compared with conventional formulations. Consequently, it could lower its residue on agricultural products and the risks to humans and the environment.^4–6^

Colloidal delivery systems show high efficiency as a means of efficiently delivering one or a mixture of active ingredients to their site of action. Furthermore, PNC can reduce the side effects of the insecticides and improve the photostability of active ingredients.^7^ A lot of work has been done on colloidal delivery systems in agricultural sector. Acephate polymeric nanocapsule synthesized with polyethylene glycol (PEG-400) showed increasing solubility in water, increasing stability and efficiency at a lower dose, reducing the economic cost for each application, and decreasing acephate toxicity to beneficial insects when compared with the commercial acephate formulation.^8,9^ Polyethylene glycol (PEG) coated nanoparticles (NPs) loaded with garlic essential oil were efficient against adult *Tribolism castaneum*.^10^ The control efficiency against adult *T. castaneum* was remained over 80% after five months with NPs loaded garlic essential oil but was only 11% with garlic essential oil alone. The EB microcapsules based on a copolymer matrix of silica–epichlorohydrin–carboxymethylcellulose could protect effectively EB against photo- and thermal degradation and thereby increase their efficacy against *Myzus persicae*.^11^

The mesoporous silica nanoparticulates (MSNs) can increase the stability, dispersity, and the controlled release of pesticide compounds under environmental condition. The photostability of avermectin was improved by the porous hollow silica nanoparticle (PHSNs) carriers entrapping it into the hollow core of the nanoparticle carriers.^12^ Also the PHSNs improved the efficacy of controllable release, photo-stability and water solubility of abamectin by modifying the porous structure of silica nanoparticles, which is useful to enhance the bioavailability and decrease the residues of pesticides.^13^ In another study, it was found that the CRFs based on MSNs were successfully used to adsorb and release imidacloprid.^14^ Also, polymeric encapsulation based Functional Nano-Dispensers (FNDs) of imidacloprid showed that the FNDs were an effective releasing materials for insecticides as compared with a standard commercial formulation, providing an acceptable level of protection for at least 10 days. Additionally, the FNDs could reduce the amount of imidacloprid required to cause similar mortality of *Diaphorina citri* as compared with the commercial formulation. Moreover, FNDs have greater potential as a cost-effective solution against a number of pests.^15^

In this work, three novel functionalized EB + NFs emamectin benzoate polymeric nanocapsules (EB + PNC), emamectin benzoate silicon dioxide nanoparticles (EB + SNPs) and emamectin benzoate mesoporous nanosilica (EB + MCM-48), were prepared based on colloidal delivery systems to improve the EB stability under various environmental conditions such as sunlight and humidity. The EB + NFs were characterized with regard to their particle size distribution, zeta potential (ZP), entrapment efficiency (EE), and morphology. Photostability studies were also performed for all EB + NFs *in vitro* condition. The toxicity of three EB + NFs against diamondback moth, *Plutella xylostella* L. (Lepidoptera: Plutellidae), were evaluated and compared with EB alone.

## 2. Experimental details

### Materials

Emamectin benzoate (70%) was kindly provided by Institute of Pesticide & Environmental Toxicology, Zhejiang University. Ethyl cellulose, sodium silicate (Na_2_SiO_3_), sulfuric acid (H_2_SO_4_), cetyltrimethylammonium bromide (CTAB), tetraethyl orthosilicate (TEOS, 98%) and ammonium hydroxide (NH_4_OH) solution were purchased from Sangon Biotech (Shanghai) Co., Ltd, (Shanghai, China). All organic solvents (methanol, ethanol, acetonitrile, acetic acid, and dichloromethane) [high-performance liquid chromatography (HPLC) grade], Pluronic® F-127 (nonionic surfactant), sorbitan monostearate (Span 60) and sorbitan monooleate (Tween 80) were purchased from Sigma-Aldrich (St. Louis, MO, USA). Ultrapure water was produced in our laboratory using a Milli-Q System (18 MΩ) (Millipore Corp., Bedford, MA, USA).

### Preparation of EB + NFs

#### Preparation of EB + PNC

The EB + PNC was prepared according to Forim's method with some modifications.^1^ Nanoemulsion was prepared by vigorous homogenization. The mixture of 0.2 g a.i. of EB dissolved in 5 mL methanol and 0.2 g of Span 60 poured in 100 mL water was stirred through Ultra-Turrax homogenizer (IKA T 10 B S25 basic Ultra-Turrax; Ika-Werke, Germany) at 28 000*g* for 5 min to produce EB nanodrops. After a brief period of stabilization, the solution of 0.5 g of ethyl cellulose dissolved in 20 mL ethanol was poured into nanodrops of EB under magnetic stirring by mini air compressor AS18BK with airbrush HS-30K (Haosheng Pneumatic Machinery Co., Ltd, Ningbo, China), under pressure 30 psi. After stirring above mixture for 10 min, the third solution of 0.2 g of Tween 80 in 20 mL water was poured to the previously made solution under magnetic stirring for 10 min.

#### Preparation of EB + SNPs

The SNPs was prepared using sol–gel technique in accordance with the Music's method with some modifications.^16^ A total of 0.2 g equivalent sodium silicate (Na_2_SiO_3_) was diluted in 300 mL ultrapure water, and 0.2 g equivalent sulfuric acid (H_2_SO_4_) was diluted in 200 mL ultrapure water. The H_2_SO_4_ solution was added drop by drop into the sodium silicate solution. The mixture was mixed through a magnetic stirrer for 45 min to form a nanosilica gel. The gel was washed six times in a filter paper with ultrapure water to remove excessive sodium sulfate (Na_2_SO_4_) from the mixture under vacuum filtration. The gel was dried by using an ALPHA 1-2 LD plus freeze dry (Martin Christ Gefriertrocknungsanlagen GmbH, Osterode am Harz, Germany) for 24 h to obtain SNPs powder.

The SNPs loaded with EB were prepared by a freeze drying technique, colloidal solution from SNPs and EB (a.i.) were mixed at a ratio of 1 : 1 by weight. Firstly, 0.8 g SNPs was diluted in 200 mL ultrapure water, and the SNPs colloidal solution was sonicated for 30 min to ensure the nanoparticles are separated from each other. Then, 0.8 g EB (a.i.) was dissolved in 20 mL methanol. The EB solution was added drop by drop into the colloidal SNPs under continuously stirred condition by the magnetic stirrer at speed 600 rpm for 2 h at room temperature. After 2 h the EB could be completely lodged on the surface of SNPs and then the mixture was dried by using the ALPHA 1-2 LD plus freeze dry for 24 h to get EB + SNPs powder.

#### Preparation of EB + MCM-48

The MCM-48 nanoparticles were prepared using the method of Kim *et al.*^17^ Briefly, 0.5 g CTAB and 2.05 g Pluronic F127 were dissolved in a solution of ultrapure water (96 mL), ethanol (34 mL) and 29% (by weight) ammonium hydroxide solution (10.05 mL) at room temperature. After complete dissolution, 1.8 g of TEOS was added into the mixture at once. After 1 min of mechanical stirring at 1000 rpm, the mixture was kept at a static condition for 24 h to generate silica nanoparticles. Resulting white precipitates were collected, centrifuged and washed twice with ethanol, and dried under vacuum. Finally the dried precipitates were calcined at 550 °C for 5 h.

Loading experiments were carried out in methanol as EB is highly soluble in methanol. EB (a.i.) and the MCM-48 carriers were mixed at a weight ratio of 2 : 1 (a.i. EB : MCM-48). The MCM-48 (0.5 g) were suspended in 100 mL methanol and sonicated for 30 min. EB (1 g a.i.) was dissolved in 50 mL methanol, then the EB solution was added drop by drop into MCM-48 suspensions. Whole solution was continuously stirred by magnetic stirrer at 600 rpm for 30 min at room temperature. The mixed solution was shaken for 24 h, a time period found to be sufficient to reach equilibrium. After 24 h impregnation, the suspensions were used to evaluate EB + MCM-48.^14^

### Determination of emamectin benzoate content

#### Determination of emamectin benzoate in EB + PNC

The total amount percentage of absolute recovery (AR%) of EB in EB + PNC was determined using the following method. First, 0.1 mL of the EB + PNC was dissolved in 0.9 mL of ethanol for two hours. After polymer dissolution, the solution was centrifuged (centrifuge 5417 R; Eppendorf, Germany) at 20 800*g* for 30 min at 20 °C. After phase separation, 0.5 mL of the supernatant was dried under vacuum (concentrator plus, Eppendorf, Germany). Then, the dried compound containing EB was dissolved in 2 mL of methanol, and the total amount of EB was determined by High Performance Liquid Chromatograph (HPLC), Agilent 1260, equipped with UV detector at 245 nm and HPLC column (Zorbax Eclipse XDBC18 (150 × 4.6 mm i.d., 5 μm particle size, stainless steel)). The mobile phase consists of acetonitrile (99.8%): acetic acid (0.1%) (80 : 20 v/v). The injection volume was 20 μL with a flow rate of 1.0 mL min^−1^, the oven temperature maintained at 30 °C.

The percentage of entrapment efficiency (EE%) of EB in EB + PNC was determined by measuring the concentration of the free unloaded compound in the aqueous phase of the EB + PNC. Centrifugation was carried out using a tube filter containing 0.22 μm pore cellulose acetate membrane (Costar Spin-X, Corning Inc.). A total of 0.5 mL of colloidal EB + PNC suspension was placed in the outer chamber of the filter assembly, and the assembly was then centrifuged at 2700*g* for 15 min at 15 °C. The encapsulated compounds were remained in the outer chamber, whereas the aqueous medium containing the free unloaded EB was moved to the sample recovery chamber through the filter membrane. After separation, 0.2 mL of the aqueous medium was dried. The dried product was dissolved in 2 mL of methanol, and subsequent concentration was determined by HPLC as described earlier by Forim *et al.*^1^ The EE% was subsequently calculated using the following equation:
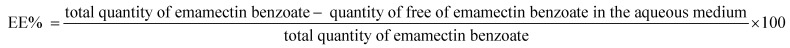


#### Determination of EB in EB + SNPs and EB + MCM-48

The total amount percentages of absolute recovery (AR%) of EB in the nanoformulations were determined using the following method. First, 1 mL of the EB + SNPs or EB + MCM-48 solution was dissolved in 10 mL of dichloromethane, and the samples were sonicated for 30 min and then the mixture was magnetically stirred at 1000 rpm for 3 h to ensure complete extraction of the EB from samples. After this, 1 mL from the samples were centrifuged (centrifuge 5417 R; Eppendorf, Germany) at 20 800*g* for 30 min at room temperature. After phase separation, 0.1 mL of the supernatant was dried under vacuum (concentrator plus, Eppendorf, Germany). Then, the dried compound containing EB was dissolved in 1 mL methanol, and the total amount of EB was determined by HPLC as done in EB + PNC.

The percentage of entrapment efficiency (EE%) of EB was determined by measuring the concentration of the free unloaded compound in the aqueous phase of the colloidal solution according to Forim *et al.* as previously mentioned method.^1^

### Characterization of EB + NFs

The surface morphology was observed using scanning electron microscope (SEM, TM-1000, Hitachi, Japan) and transmission electron microscope (Tecnai™ Spirit TEM, FEI Company, Hillsboro, OR, USA). X-ray diffraction (XRD) measurements were performed using a multipurpose X-ray diffractometer (XRD, X'Pert PRO, PANalytical, Almelo, The Netherlands) with Cu radiation (*λ* = 1.54 A) at 40 kV and 40 mA. The samples were scanned from 1 to 90° *θ*, and the scanning rate was 2° *θ* per min with step size of 0.02° *θ*. Fourier transform infrared spectrophotometer (FT-IR) (Vector 22, Bruker, Germany) was used to identify the different functional groups presented in the samples. Particle size and zeta potential values were evaluated by NanoPlus “Particle Size & Zeta Potential Analyzer” (Particulate Systems a division of Micromeritics, 4356 Communications Drive, Norcross GA, 30093, USA).

### Stability assay of EB + NFs against UV radiation

The stability of the EB + NFs against ultraviolet (UV) radiation was tested by exposing the samples to a 36 W germicidal lamp (254 nm) at a distance of 20 cm at room temperature. The samples were withdrawn every 12 h in the 72 h for analysis and the changes of the EB content were analyzed by HPLC. The methanol solution of the EB (a.i.) was used as the control sample at the same time.^11^

### Release study

The release experiments were carried out according to the method of Guo *et al.*^11^ 0.1 g from different EB + NFs were weighed and suspended in 100 mL of the methanol–water mixture (30 : 70, v/v). This methanol–water mixture was used as a release medium in order to dissolve the EB. 5 mL suspension of the EB + NFs was introduced into a dialysis bags and stirred at a speed of 100 rpm at room temperature, then the released EB from the dialysis bags was monitored up to 72 h. The released solution was collected at different intervals after (2, 4, 6, 12, 24, 36, 48, 60 and 72 h), and centrifuged at speed 10 000 rpm for 15 min at room temperature. The concentration in the supernatant was determined using HPLC and the cumulative release rate of the EB from the EB + NFs was calculated to evaluate the sustained release properties. Data of the gradual release curve from EB + NFs release experiments were fitted to the following equation.^18,19^
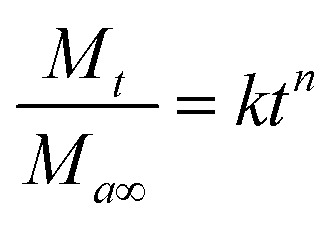
*M*_*t*_ is the amount of EB released at time *t*, *M*_*a*∞_ is the total amount of EB in EB + NFs, *k* is a release constant and *n* is a diffusional exponent.

### Insect culture

The initial population of *P*. *xylostella* was collected from a cruciferous vegetable field in the eastern suburbs of Hangzhou (30°14′N, 120°15′E), Zhejiang Province, China, in September 2014. *P. xylostella* was reared at 25 ± 2 °C with 70% ± 10% relative humidity (RH) and a light–dark cycle of 16 : 8 h. The larvae were reared on cabbage leaves (*Brassica oleracea* var. capitata (L.) (*Capparales*: *Brassicaceae*) cv. Jingfeng No. 1), and adults were fed with 10% sucrose solution.^20^

### Bioassay experiments

The bioassay for the efficacy of EB + NFs against the early third instar larvae of *P. xylostella* was performed in plastic containers (14.2 × 7.2 × 5.2 cm) using leaf dipping method and carried out at 25 ± 2 °C with 70% ± 10% RH and a light–dark cycle of 16 : 8 h. Dilutions of EB + NFs and EB were prepared using ultrapure water. The concentrations were prepared based on the mortality rang falling between 20% and 80%,^21^ and the total volume was 100 mL for each concentration. Leaf discs (2.5 cm in diameter) of cabbage leaves were dipped for 30 s in the test solution with gentle agitation. The leaf discs dipped in ultrapure water were served as control. Thirty min later, the surface of leaf discs was air dried, one dipped leaf disc with 30 early third instar larvae was placed in a perforated plastic container. The larvae were allowed to feed on the treated leaf disc for 24 h, and then fed them with clean leaf disc until the end of the experiment. Insect mortality was recorded at 24, 48, and 72 h after the larvae were exposed to EB + NFs or EB alone. Three replicates for each concentration were performed, and 30 larvae were utilized for each replicate.

### Statistical analysis

The mortality was analyzed *via* a two-way analysis of variance (ANOVA) with IBM SPSS Statistics 19 software. All percentage data were transformed using arcsine square root before ANOVA to standardize means and normalize variances and were transformed back to percentage for presentation. Mean values were separated through the least significant difference (LSD) test (*P* < 0.05) when significant differences among several mean values were detected by ANOVA. The LC_50_ values were calculated using the statistical method of LdP line program software (http://embakr.tripod.com/ldpline/ldpline.htm), which was devoted to the calculation of probit analysis based on Finney's method.^22,23^

## 3. Results and discussion

### Encapsulation efficiency of EB + NFs

**Fig. 1:**
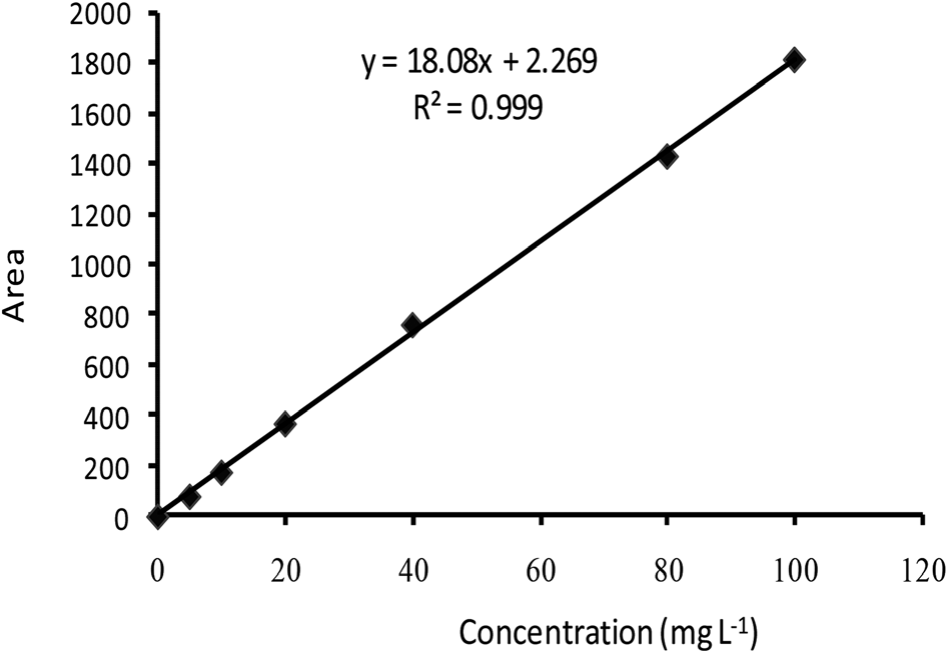
Standard curve of emamectin benzoate.

The standard curve of EB showed a good linear relationship between the concentrations that ranged from 5 to 100 mg L^−1^ with linear equation *y* = 18.08*x* − 2.2698 (*R*^2^ = 0.9994) (Fig. 1). The AR% was 72.51%, 94.55% and 74.29%, and EE% of EB + NFs was 92.84%, 87.45% and 71.19% from EB + PNC, EB + SNPs and EB + MCM-48, respectively (Table 1).

**Table 1:** Characterization of EB + NFs

Formulation	Absolute recovery AR (%)	Entrapment efficiency EE (%)	Particle size (nm) ± SD	Zeta potential (mV) ± SD
EB + PNC	72.51	92.84	219.93 ± 3.89	−26.43 ± 2.90
EB + SNPs	94.55	87.45	142.77 ± 3.43	−41.00 ± 1.31
EB + MCM-48	74.29	71.19	119.73 ± 20.28	−36.50 ± 0.56

The ethyl cellulose, SNPs and MCM-48 were used to improve the EB formulations to avoid the disadvantage and the side effects of pesticides. According to the obtained EE% results, the NFs preparation method is suitable for preparation of EB + NFs.

Ethyl cellulose that are used in preparation of EB + PNC showed a higher encapsulation rate than other nanocapsule formulations, this may be due to its physical and chemical properties and its high ability to encapsulate the active ingredient in the nanocapsules.^24,25^

The EE% of MCM-48 for EB reached to 71.19%. The efficacy of MCM-48 for EB adsorption is due to its large surface area, porous structure and small particle size. The pore diameter also plays an important role in the loading process. The MCM-48 channels porous diameter is larger than the sectional diameter of EB molecules, allowing EB molecules to be entrapped into the porous structure of MCM-48 nanosilica. Furthermore, the type of MCM-48 nanosilica has a 3D cubic mesoporous structure with open networks and high surface area.^14^ This structure increase its adsorption capacity of the EB. Our results support several earlier studies.^13,14,26,27^ The amount of avermectin encapsulated in the MSNs reached 58.3% w/w by a simple immersion loading method, thus most of the adsorption of avermectin on the mesoporous nanosilica might be physical.^26^ The MSNs with a shell thickness of ∼15 nm and a pore diameter of 4–5 nm have an encapsulation capacity of 625 g kg^−1^ for avermectin using a supercritical fluid loading method.^27^ Similarly, MCM-48 nanospheres can be utilized as an effective delivery carrier owing to their high surface area and unique 3D open pore structure.^14^ In addition, the MSNs showed excellent pesticide loading capacity and delivery performance in controlled release, anti-photolysis, and water dispersity of abamectin.^13^

Pesticide loading can be achieved by different methods on the nanoparticles carriers such as extrusion, spray dry and freeze dry.^28^ A successful colloidal delivery system should have a high loading capacity. In this study, the EE of EB + SNPs reached to 87.45%, the high loading capacity maybe due to the competition between its solubility in the water and its adsorption on to the SNPs surface. Also because the EB is poorly soluble in aqueous media and its ability to be adsorbed on the SNPs is higher than its solubility in the water. In addition, silica gel is a widely employed compound in the column chromatography as a stationary phase and as adsorbents in the environmental studies because it has high adsorption potential of organic and inorganic compounds.^29^

### Characterization of the EB + NFs

#### Analyses of morphology and sizes of EB + NFs

**Fig. 2:**
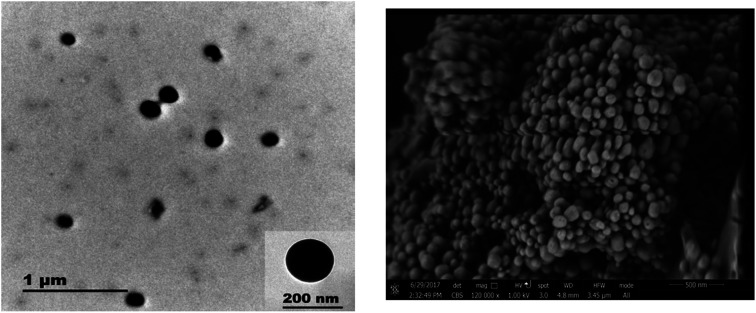
TEM (left) and SEM (right) image of EB + PNC.

**Fig. 3:**
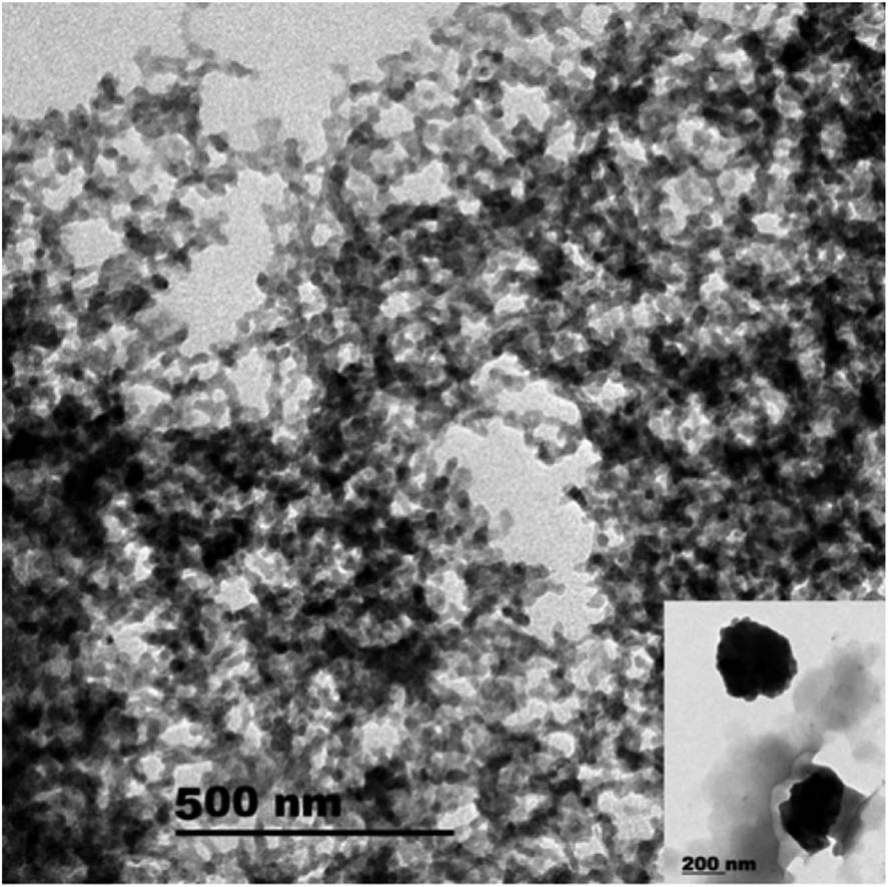
TEM image of SNPs.

**Fig. 4:**
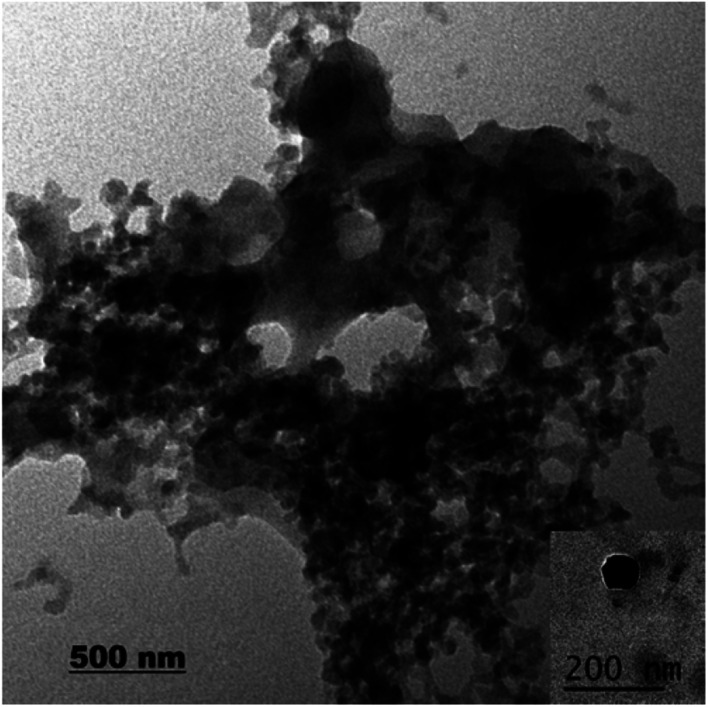
TEM image of EB + SNPs.

**Fig. 5:**
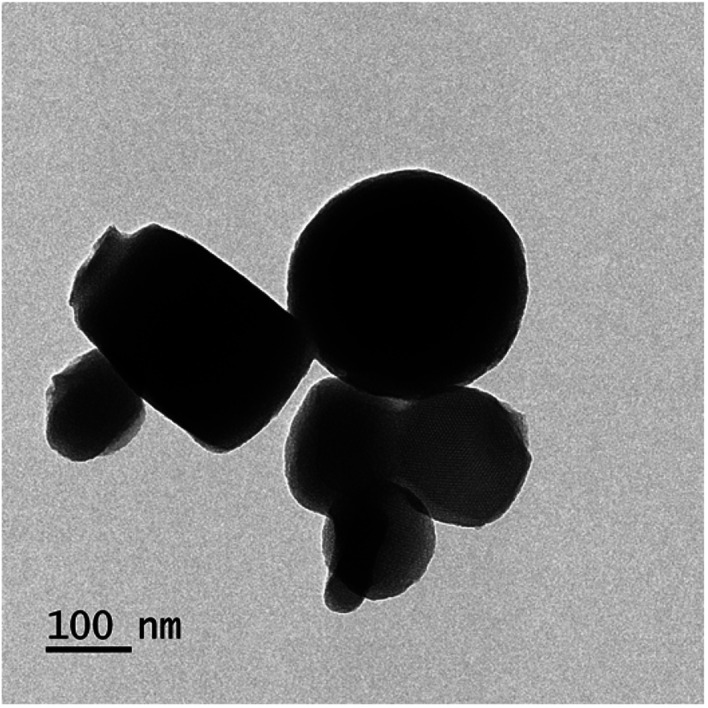
TEM image of MCM-48.

**Fig. 6:**
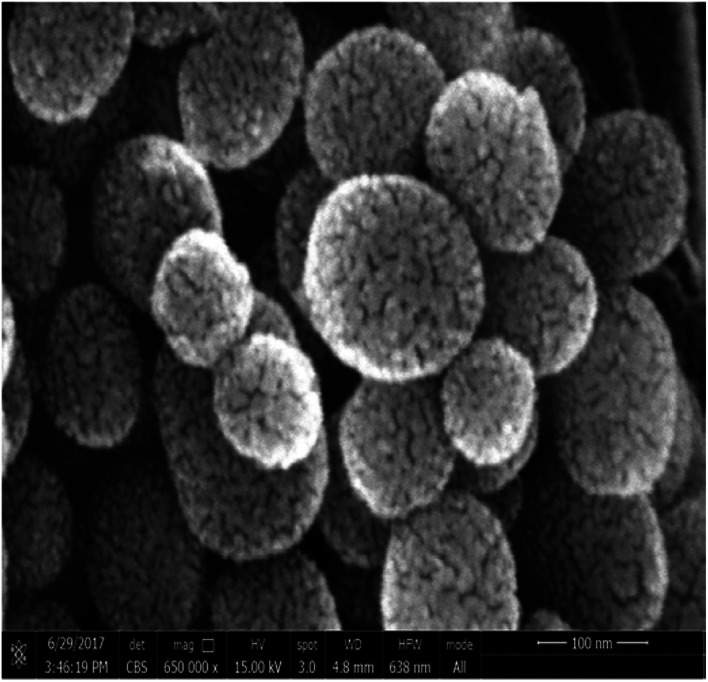
SEM image of EB + MCM-48.

Regarding to EB + PNC, according to TEM micrographs, the EB has been successfully encapsulated in the nanocapsules of ethyl cellulose (Fig. 2) and the nanocapsules were of spherical in shape with an average size of 219.93 ± 3.89 nm (Table 2). SEM micrographs also revealed that the EB + PNC had a homogeneous distribution of particles (Fig. 2). Regarding to EB + SNPs, TEM image showed that the SNPs had nanometric sizes (Fig. 3). The SNPs had spherical shape with a small percentage of irregular surfaces, TEM image showed that the EB adsorbed by SNPs and created thin film around the SNPs (Fig. 4), and the average size was around 142.77 ± 3.43 nm (Table 1). The TEM image of the MCM-48 is presented in Fig. 5. The TEM micrographs showed that core–shell-structured silica nanoparticles, with an average size of 119.73 ± 20.28 nm (Table 1) and a shell thickness of 10 ∼ 15 nm. The EB was incorporated in pores of MSNs to obtain controlled release formulation. The pore size of MCM-48 wall is larger than the diameter of EB, allowing EB molecules to be entrapped into the MCM-48 (Fig. 6).

**Table 2:** Analysis of variance of the main parameters and their interactions

Source	EB		EB + PNC		EB + SNPs		EB + MCM-48	
	*F* value	*P* value	*F* value	*P* value	*F* value	*P* value	*F* value	*P* value
Time	259.62	0.0001	428.39	0.0001	288.55	0.0001	77.57	0.0001
Conc.	392.98	0.0001	580.83	0.0001	1776.89	0.0001	221.82	0.0001
Time* conc.	21.12	0.0001	32.36	0.0001	20.07	0.0001	4.8	0.0001

The well-known ethyl cellulose is one of the most constructive polymer, and is used widely to synthesize the microcapsules and nanocapsules for drugs and pesticides because of its advantages as formulator, such as perfect film formability, higher physical–chemical stability, and minimum toxicity.^24,25,30,31^ Regarding MCM-48, the MCM-48 has an average diameter of 119.73 ± 20.28 nm with a spherical in morphology and a highly organized porous structure, and it has similar characteristic reported by Popat *et al.*^14^

The freeze-drying technique is used to enhance the stability of colloidal nanoparticles which also include nanocarriers for CRFs of pesticides. It is widely used for drying the unstable or heat-sensitive compounds at low temperatures without damaging their chemical structure. In this study, the SNPs colloidal solution mixed with EB (a.i.), then the aqueous phase was frozen and subjected to a low-pressure system. When the pressure is reduced drastically, the water sublimates (goes from solid to vapor state) and the EB a.i. leaves on SNPs.

#### Regarding to XRD analysis

**Fig. 7:**
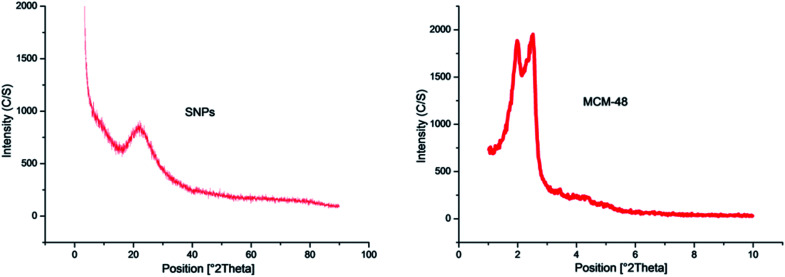
XRD pattern of SNPs and MCM-48 nanosilica.

It was used to investigate the structure of SNPs and MCM-48 nanoparticles. The XRD patterns of SNPs and MCM-48 were showed in (Fig. 7). The SNPs peak was observed at 21.218° *θ* using Bragg's law, that is, *λ* = 2*d* Sin *θ*, and the MCM-48 peak was observed at 2.6° *θ*. These results showed a broad peak for an amorphous nanosilica core region. The XRD pattern of SNPs was compatible with earlier studies which reported that the peak of SNPs was approximately at 20° *θ*.^32,33^ Regarding to MCM-48 nanosilica, the obtained results established that the synthesized compound is MCM-48 nanosilica. This result was in accordance with Choi *et al.,*^34^ they observed a sharp first Bragg peak indexed as (211) at 2° *θ* = 2.51° *θ* and the second peak (220) at 2.89° *θ* for the cubic *Ia*3*d* mesostructure (MCM-48). It is also compatible with previous studies which discussed the chemical and physical characterization for the MCM-48 nanosilica.^35–37^

#### FT-IR analysis

**Fig. 8:**
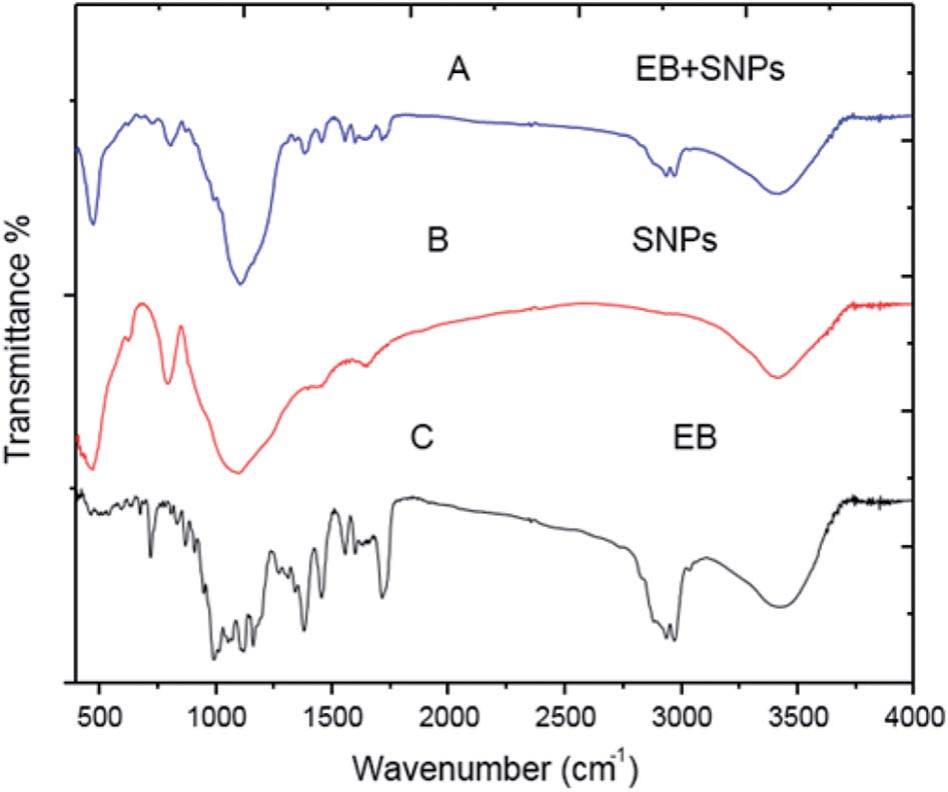
FTIR spectrum of SNPs and EB + SNPs.

**Fig. 9:**
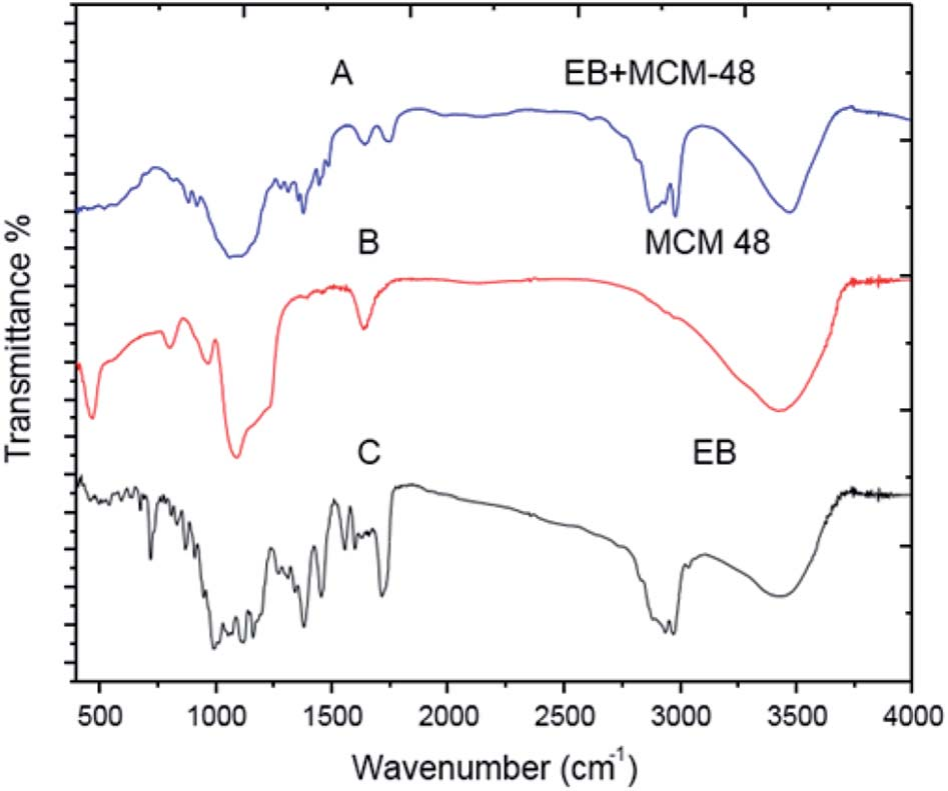
FTIR spectrum of MCM-48 and EB + MCM-48.

The infrared spectra of EB blank, SNPs, MCM-48, EB + SNPs and EB + MCM-48 samples showed in Fig. 8 and Fig. 9. Regarding to SNPs, the absorption band at 3419 cm^−1^ (Fig. 8 A) showed that only a small amount of water is present in the samples. The very broad strong peak at 1097 cm^−1^ can be ascribed to composite of Si–O stretching of nanosilica (Fig. 8 B). For the free EB, the bands at 2967 and 2982 cm ^−1^ are attributed to C–H stretching vibrations of an aromatic ring corresponding to the benzoate fraction or conjugated olefins, 1716 cm^−1^ bending vibration of (C=O stretching vibrations of an acrylics ester), 1634, 1599 and 1557 cm^−1^ are ascribed to (C=C stretching vibrations of an aromatic ring or conjugated olefins), 1455 and 1379 cm^−1^ are identified as skeleton vibration of (C–H deformation in CH3 groups), 1160, 1118 and 1058 cm^−1^ are attributed to (C–O stretching vibrations, O–H and C–O–C flexion), 991 cm^−1^ bending vibration of (C–H flexion of trans C=C bonding) and 947–568 cm^−1^ (C–H flexion outside the plane in an aromatic ring or C=C cis bond) (Fig. 8C). For the EB + SNPs, the spectrum retained most of the major peaks of SNPs and EB, and no noticeable new peaks were observed in EB + SNPs (Fig. 8A). The FT IR of free EB blank, MCM-48 and EB + MCM-48 showed in Fig9. The obtained results for blank MCM-48 were similar with SNPs spectrum that mentioned above, and the FT IR of EB, was mentioned above (Fig. 9C). The very broad strong peak at 1090 cm^1^ can be ascribed to composite of Si–O stretching of nanosilica (Fig. 9B) and no noticeable new peaks were observed in EB + MCM-48 (Fig. 9A).

The FT-IR analysis was employed to examine possible interactions between the EB and the nanosilica carriers. The FT-IR results confirmed that the EB + NFs spectrums retained most of the major peaks of pure carriers (SNPs and MCM-48) and EB, and did not show noticeable new peaks, indicating that the adsorption of EB in the NPs carriers is probably physical adsorption. Therefore, the properties of EB have not been changed after their loading on the NPs carriers. These results were convenient with the absorption behavior of avermectin–PHSN as reported by Wen *et al.*^26^

#### Zeta potential

The zeta potential degree describes the electrostatic repulsion degree between adjacent, similarly charged particles in dispersion. The colloidal solution with ZP of > +30 mV or < −30 mV is considered to be very stable. The zeta potential values for all EB + NFs were fallen in the range of −26 to −41 mV (Table 1). Zeta potential is an excellent technique for describing the properties of the nanoparticle surface and predicting the long term existence of the nanoparticle. In the low zeta potential, attractive forces may overcome this repulsion and the dispersion may split and flocculate. So, the colloidal system with high zeta potential are electrically stabilized while the colloidal system with low zeta potentials are electrically unstable.^38,39^ Especially, the NPs with zeta potential of > +30 mV or < −30 mV is considered to be very stable in the dispersion medium.^1,40^ In our study, the zeta potential values for all EB + NFs were more than −26 mV, suggesting that the NPs formulations were stable in the dispersion medium.

#### UV stability

**Fig. 10:**
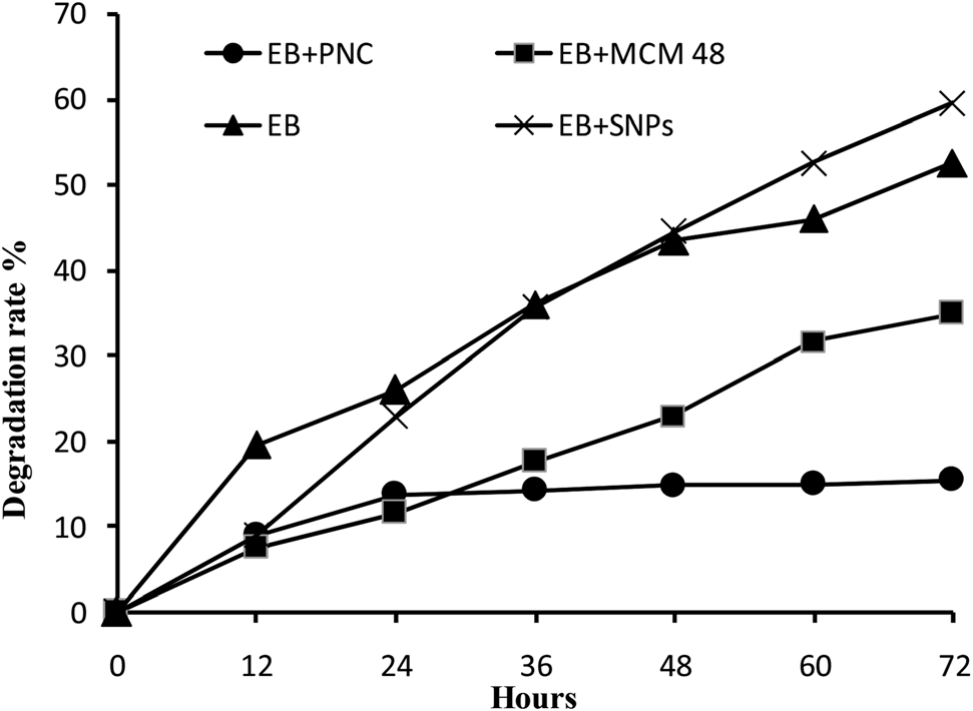
Stability of the resulting EB + NFs and the active ingredient against UV radiation.

The photo-stability of EB + NFs and the EB alone showed in Fig. 10. The degradation rates were 15.35, 34.94, 52.58 and 59.50% for EB + PNC, EB + MCM-48, EB and EB + SNPs after exposure of 72 h, respectively. These results showed that the EB + PNC was more stable than all other samples, and no significant difference was detected between EB and EB + SNPs. The UV radiation results showed that the EB could be protected through the EB + PNC and EB + MCM-48, nanosilica wall can significantly prevent the photolysis of EB and increase the EB stability. While the EB + SNPs did not protect EB from photolysis because EB was only adsorbed on the surface of SNPs.

Protecting the a.i. of a formulation under field conditions is necessary when the local environment adversely affects the stability of the pesticides. So, the encapsulation is necessary to overcome the stability problems of a.i. and also to improve the solubility of this pesticide in water.^28^

With regard to EB + PNC, the ethyl cellulose enhanced the photostability of EB + PNC, the stability against UV radiation maybe due to the physical and chemical characteristics of ethyl cellulose. The ethyl cellulose has been widely used for microencapsulation due to its versatile properties such as melting point range 240–255 °C, specific density range 1.07–1.18 with 135–155 °C heat distortion point and 330–360 °C fire point, stability against light, heat, wetness and chemicals, and ability to absorb pressure.^41^ Because, the polymeric chain forms a stronger film isolating the a.i. from the external environment, it can protect the a.i. from the degradation by UV. The previous studies confirmed that the polymeric nanocapsules could improve the persistence of a.i. against UV radiation such as natural products,^1^ acetamiprid microcapsules^4^ and the EB slow release microspheres.^42^ Consequently, the polymeric nanocapsule is able to protect the a.i. from the rapid degradation or may increase the efficiency of pest control for a long duration. Furthermore, it can be able to lower the dosage of pesticides and exposure to human.

The EB + MCM-48 reduced significantly the degradation rate of EB. This is probably due to the EB was entrapped into the pores of MCM-48. These results were consistent with the previous study by Guo *et al.*,^11^ they found that the EB was sensitive to UV radiation and the samples were degraded completely within 48 h, while the decomposition rate of the EB wrapped in microcapsules was less than 25% after 72 h of UV exposure. On the other hand, the MSNs highly improved the photostability of avermectin by entrapping it into the hollow core of the nanoparticle carriers.^12,26^ The MSNs not only can protect the active ingredient from UV radiation but also can enhance the chemical solubility and its dispersity in the water.^13^ Improvement of the insecticides stability can reduce the concentration of insecticides in commercial spray applications, without lowering the efficiency. These kinds of the formulations (EB + PNC and EB + MCM-48) are convenient for application in the early stage of plant life, which requires stable pesticides under various environmental conditions to protect the plant for long period. Consequently, they may reduce economic cost by decreasing the number of applications. Moreover, insecticide nanodelivery systems were proposed to increase the spatial distribution on leaf surfaces of crops due to the nanosize and thereby enhance the effectiveness of pesticide applications. Besides, pesticide nanodelivery systems also have better penetration ability through the cuticle, and allow slow and controlled release of active ingredients on the target.^13^

The EB and EB + SNPs showed no significant difference in the stability against UV radiation. The SNPs do not has the ability to protect the a.i., this may be due to that the active ingredient in EB + SNPs were adsorbed on the surface of the SNPs, and exposed directly to UV radiation.

#### The release behaviors of EB + NFs

**Fig. 11:**
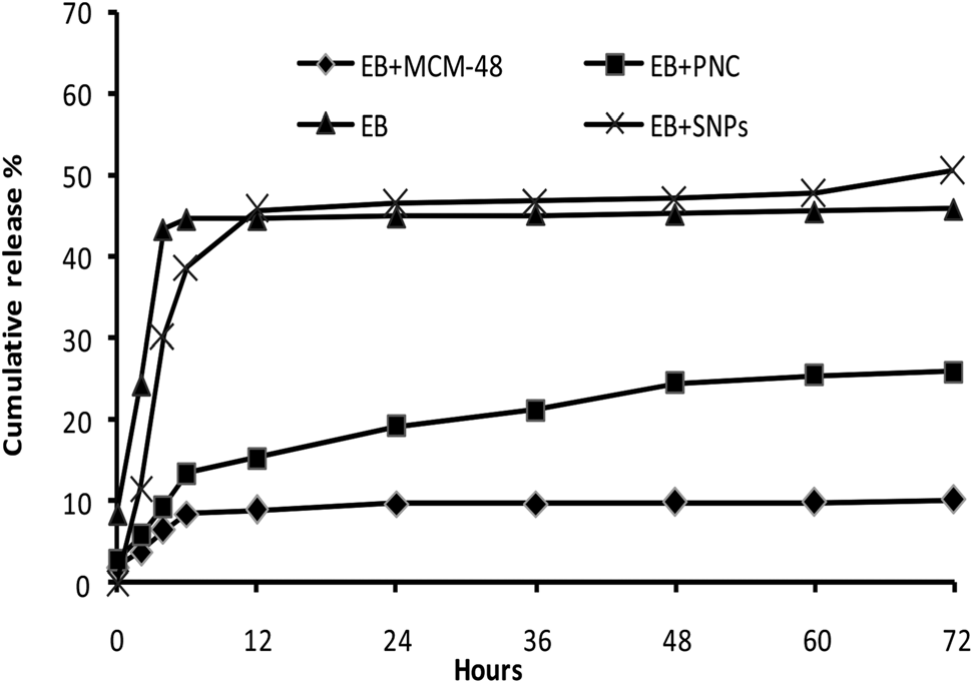
The release behaviors of the EB and EB + NFs.

The release profiles of EB + NFs showed in the Fig. 11. The releases of EB in all samples were relatively fast at initial stage and then progressively slow with increasing time. The cumulative release rates were 10.11, 25.84, 45.77 and 50.47% after 72 h for EB + MCM-48, EB + PNC, EB and EB + SNPs, respectively.

CRFs play a critical role to reduce the environmental problems associated with the application of pesticides. The colloidal delivery system has great potential to improve the pesticide CRFs and remarkably reduce effective dosage by maintaining an effective concentration in the target for longer periods of time.^13^ In this study, the EB + MCM-48 showed two stages in release behavior. The release in the first few hours was so fast, then, the release rate became much slower in the later hours. In the first stage, it may be due to the dissolvent of the EB adsorbed on the external surface of MCM-48, while in the second stage, it may be due to the hindrance of the porous structure of MCM-48. These results showed good agreement with the release behaviors of avermectin porous hollow silica nanoparticles (avermectin-PHSNs), abamectin-PHSNs and imidacloprid-mesoporous silica nanoparticles formulations.^13,14,26^

The EB + PNC release behavior was similar to that of EB + MCM-48 release. The slow release profile may be due to the stronger mechanical property of the nanocapsules that prevent the excessive release of EB from the EB + PNC. Moreover, the denser ethyl cellulose polymer chains and its physicochemical properties led to the reduction in release.^24,25^ The release profiles of EB both in EB + SNPs and in the control (EB alone) showed no significant difference. Both showed a faster release at first 12 h, this may be due to the fast dissolution of EB on the surface of SNPs.

#### Bioassay

All main effects and their associated interactions were significant at *P* < 0.0001 level (Table 2). In all treatments, mortality percentage increased with the increase in concentrations and with the passage of time (Table 3). The efficacy of EB + NFs was compared on the LC_50_ values. Results showed that the EB + SNPs is more effective than other treatments, because it showed 100% mortality after 48 h but other treatments EB + MCM-48, EB and EB + PNC showed 86.67%, 88.86% and 84.44% mortality after 72 h respectively (Table 3). The lowest LC_50_ was recorded when larvae were exposed to EB + SNPs, followed by EB + MCM-48 which are much lower than EB + PNC and EB. However, no significant difference was observed between the EB + PNC and the EB. The LC_50_ value of EB + SNPs was 0.18 mg L^−1^ after 48 h, while the LC_50_ values for other treatments were 1.8, 4.22 and 4.52 mg L^−1^ of EB + MCM-48, EB and EB + PNC after 72 h, respectively (Table 4).

**Table 3:** Mortality (mean ± SD) of the third instar larvae of *P. xylostella* after EB + NFs exposure *via* leaf dipping^a^

Formulation	Concentration (mg L^−1^)	Hours after treatment		
		24 h	48 h	72 h
EB + SNPs	0.1	14.77 ± 1.92 e	27.27 ± 1.92 e	34.09 ± 1.92 c
	0.2	31.81 ± 3.33 d	52.27 ± 3.33 d	65.91 ± 3.33 b
	0.4	62.43 ± 3.33 c	79.54 ± 3.33 c	100.00 ± 0.00 a
	0.8	76.14 ± 3.33 b	86.36 ± 3.33 b	100.00 ± 0.00 a
	1.6	86.36 ± 3.33 a	100.00 ± 0.00 a	100.00 ± 0.00 a
	Control	0.00 ± 0.00 f	0.00 ± 0.00 f	0.00 ± 0.00 d
EB + MCM-48	0.5	10.00 ± 1.92 d	13.33 ± 3.33 d	22.22 ± 5.09 e
	1	14.44 ± 1.92 cd	21.11 ± 3.85 cd	38.89 ± 5.09 d
	2	21.11 ± 5.09 c	26.67 ± 6.67 c	47.78 ± 5.09 c
	4	37.78 ± 5.09 b	45.56 ± 3.85 b	64.44 ± 6.94 b
	8	53.33 ± 8.82 a	72.22 ± 10.18 a	86.67 ± 3.33 a
	Control	0.00 ± 0.00 e	0.00 ± 0.00 e	0.00 ± 0.00 f
EB + PNC	1	4.44 ± 0.00 cd	11.11 ± 3.33 d	17.77 ± 3.33 d
	2	5.56 ± 1.92 c	14.44 ± 1.92 d	26.67 ± 1.92 d
	4	8.89 ± 1.92 c	25.55 ± 3.33 c	41.11 ± 3.33 c
	8	17.78 ± 1.92 b	43.33 ± 1.92 b	60 ± 3.85 b
	16	37.78 ± 3.85 a	73.33 ± 3.85 a	88.86 ± 3.83 a
	Control	0.00 ± 0.00 e	0.00 ± 0.00 e	0.00 ± 0.00 e
EB	1	5.56 ± 1.92 de	11.11 ± 1.92 d	16.67 ± 3.33 e
	2	8.89 ± 3.85 cd	14.44 ± 1.92 d	24.44 ± 1.92 d
	4	13.33 ± 3.33 c	23.33 ± 3.85 c	51.11 ± 3.85 c
	8	24.44 ± 1.92 b	41.11 ± 1.92 b	67.78 ± 5.09 b
	16	42.22 ± 3.85 a	62.22 ± 5.09 a	84.44 ± 5.09 a
	Control	0.00 ± 0.00 e	0.00 ± 0.00 e	0.00 ± 0.00 f

a Different letter in column under same formulation followed after mean (±SD) indicate significant difference at *P* = 0.05 level.

**Table 4:** LC_50–90_ values of EB + NFs against the third instar larvae of *P. xylostella*^a^

Formulation	Time (h)	LC_50_ (mg L^−1^) (LCL–UCL)	LC_90_ (mg L^−1^) (LCL–UCL)	Slope ± SE	*x* ^2^
EB + SNPs	24	0.32 (0.27–0.37)	1.67 (1.26–2.47)	1.78 ± 0.16	2.66
	48	0.18 (0.15–0.21)	0.75 (0.60–0.99)	2.07 ± 0.19	3.47
EB + MCM-48	24	7.44 (5.37–12.24)	89.03 (40.26–347.38)	1.18 ± 0.16	1.23
	48	4.03 (3.23–5.34)	33.81 (19.96–76.32)	1.38 ± 0.16	5.05
	72	1.80 (1.46–2.21)	14.27 (9.61–25.64)	1.42 ± 0.15	3.32
EB + PNC	24	34.79 (21.51–82.29)	359.51 (131.92–2392.08)	1.26 ± 0.22	3.23
	48	8.49 (7.00–10.74)	55.23 (35.88–103.62)	1.57 ± 0.16	3.82
	72	4.52 (3.83–5.37)	26.30 (19.05–41.02)	1.67 ± 0.15	7.45
EB	24	24.83 (17.41–56.21)	311.32 (119.88–1783.47)	1.20 ± 0.18	1.32
	48	11.06 (8.56–15.75)	99.64 (53.95–265.99)	1.34 ± 0.16	2.70
	72	4.22 (3.55–5.04)	24.14 (17.46–38.08)	1.69 ± 0.16	1.70

a LCL: lower confidence limit and ULC: upper confidence limit.

The improved efficacy of EB + SNPs and EB + MCM-48 may be due to the smaller particle size, higher surface area and their high mobility ratio, which eventually lead to increasing penetration of NPs formulations in the larval body than the active ingredient alone. The surface-functionalized silica nanoparticles can deliver DNA and drugs into animal cells and tissues,^43^ because nanoparticles drug carriers have the potential to cross physiological barriers and access different tissues.^44^ The insecticidal activity of pyridalyl nanosuspension was more effective than the commercial formulation and was 2.26 and 6.25 times more effective against *H. armigera* as stomach poison than the technical product and commercial formulation respectively.^45^ They thought that the increased toxicity of nano sized formulation on larvae is probably due to increasing penetration of pyridalyl in the larval body.

Regarding to the effectiveness of EB + PNC and EB, a little difference was noticed between their effectiveness. At the equal concentration, EB showed more effective than that of the EB + PNC in the first day, whereas EB + PNC was more effective than EB alone during the second day. These results may be due to the efficacy of EB + PNC is dependent on the controlled release of the a.i. from the nanocapsules. These results are consistence with the findings of Guo *et al.*,^11^ who reported that when treated *M. persicae* at the same concentration, EB 1% EC is more effective than that of the microcapsules at first day after treatment. Similar results were also reported by Zhang *et al.*, where the effectiveness of phoxim microcapsules increased with the passage of times.^46^ The similar results could be due to that the insecticides were loaded on the similar carrier with similar physicochemical properties.

## 4. Conclusions

In conclusion, according to our study, we can suggest that the colloidal delivery systems such as SNPs, MCM-48 and PNC could act as a controlled release carrier and can maintain chemical stability of EB. They may overcome environmental sensitivity and poor water solubility, and increase the efficacy of insecticides. These advantages could eventually lead to minimize the dosage of pesticides needed, reducing the number of applications required in comparison to conventional formulations and decreasing pesticides release in the environment. However, there are necessaries to study the safety issues regarding pesticide nanoformulations on the beneficial insects and human health.

## Conflicts of interest

No potential conflict of interest was reported by the authors.

## Supplementary Material
